# Cloning and Expression of Genes for Biodegrading Nodularin by *Sphingopyxis* sp. USTB-05

**DOI:** 10.3390/toxins11100549

**Published:** 2019-09-20

**Authors:** Qianqian Xu, Hongfei Ma, Jinhui Fan, Hai Yan, Haiyang Zhang, Chunhua Yin, Xiaolu Liu, Yang Liu, Huasheng Wang

**Affiliations:** 1 School of Chemistry and Biological Engineering, University of Science and Technology Beijing, Beijing 100083, China; qianqianxu@ustb.edu.cn (Q.X.); s20180892@xs.ustb.edu.cn (H.M.); s20170900@xs.ustb.edu.cn (J.F.); zhanghy@ustb.edu.cn (H.Z.); chyin@ustb.edu.cn (C.Y.); xiaoluliu@ustb.edu.cn (X.L.); liuyang@ustb.edu.cn (Y.L.); 2 School of Architectural and Surveying & Mapping Engineering, Jiangxi University of Science and Technology, Ganzhou 341000, China; hswang1@jxust.edu.cn

**Keywords:** nodularin, biodegradation, pathway, mlrA, enzyme, activity site

## Abstract

Biodegradation is efficient for removing cyanobacterial toxins, such as microcystins (MCs) and nodularin (NOD). However, not all the microbial strains with the microcystin-biodegrading enzymes MlrA and MlrC could biodegrade NOD. Studies on genes and enzymes for biodegrading NOD can reveal the function and the biodegradation pathway of NOD. Based on successful cloning and expression of the *USTB-05-A* and *USTB-05-C* genes from *Sphingopyxis* sp. USTB-05, which are responsible for the biodegradation of MCs, the pathway for biodegrading NOD by these two enzymes was investigated in this study. The findings showed that the enzyme USTB-05-A converted cyclic NOD (*m*/*z* 825.4516) into its linear type as the first product by hydrolyzing the arginine and Adda peptide bond, and that USTB-05-C cut off the Adda and glutamic acid peptide bond of linearized NOD (*m*/*z* 843.4616) and produced dimeric Adda (*m*/*z* 663.4377) as the second product. Further, based on the homology modeling of enzyme USTB-05-A, site-directed mutants of *USTB-05-A* were constructed and seven crucial sites for enzyme USTB-05-A activity were found. A complete enzymatic mechanism for NOD biodegradation by USTB-05-A in the first step was proposed: glutamic acid 172 and histidine 205 activate a water molecule facilitating a nucleophilic attack on the arginine and Adda peptide bond of NOD; tryptophan 176 and tryptophan 201 contact the carboxylate side chain of glutamic acid 172 and accelerate the reaction rates; and histidine 260 and asparagine 264 function as an oxyanion hole to stabilize the transition states.

## 1. Introduction

Cyanobacteria are widespread in freshwater, brackish water, estuarine [1], and marine environments [2] naturally. However, cyanobacteria cause serious environmental hazards by growing excessively and producing various kinds of cyanobacterial toxins, especially microcystins (MCs) and nodularin (NOD) [3,4,5]. With water eutrophication and global warming, cyanobacterial blooms occur increasingly all over the world [4], including New Zealand [6], Australia [1], South Africa [7], the Baltic Sea [8] and the Mediterranean region [9]. Until now, more than 100 analogues of MCs and about nine analogues of NODs [10,11,12] have been described in the literature. Toxic cyanobacteria frequently result in human health hazards and wildlife fatalities [13,14,15,16,17,18,19,20]. *Nodularia spumigena* was firstly reported in the literature as the toxic cyanobacterium for livestock [21]. NOD is a monocyclic pentapeptide hepatotoxin isolated from *N. spumigena* [6] and structurally similar to MCs [22]. NOD is cyclo-(-d-erythro-β-methylAsp-(iso-linkage)-l-Arg-Adda-d-Glu-(iso-linkage)-Mdhb), where Adda is a particular C20 β-amino acid that is only found in cyanobacterial toxins and Mdhb is N-methyldehydrobutyrine. The median lethal dose (LD_50_) value of NOD for mice is about 60 μg kg^−1^ body weight [6]. Besides the acute toxicity, NOD is also a liver carcinogen that can initiate and promote tumors [23,24]. Owing to its cyclic structure with several specific amino acids [25], NOD is stable and resistant to degradation in most physical and chemical situations, such as boiling, oxidation, or hydrolysis at neutral pH [26]. 

Biodegradation is considered to be an effective solution for the removal of cyanobacterial toxins, which is also not harmful to natural environment. Many studies were reported on the biodegradation of MCs [27,28,29,30,31,32,33,34]. Bourne et al. have detected the gene cluster for MC biodegradation, which includes *mlrA*, *mlrB*, *mlrC*, and *mlrD* from *Sphingomonas* sp. strain MJ-PV. MlrA was likely a metalloprotease with an active center of H^260^AIH^263^NE^265^ that was a variant of the zinc-binding motif (HEXXH) typically found in metalloproteases [27,35]. Mutant experiments of MlrA showed that the point mutants H260A and E265A had no activity for MC-LR biodegradation [36]. Some strains of *Brevibacterium* sp. [37], *Paucibacter toxinivorans* [38] and *Sphingomonas* sp. [39] isolated in different areas have been reported to be able to biodegrade both MCs and NOD. However, not all strains with the MC biodegradation enzymes MlrA and MlrC could biodegrade NOD [33,39,40], and less information is provided on the mechanism of the enzymes for NOD biodegradation.

We isolated and identified a promising strain of *Sphingopyxis* sp. USTB-05 (GenBank accession number of the 16S rDNA sequences: EF607053) for MC-RR, MC-LR and MC-YR biodegradation. We found that an initial concentration of 42.3 mg L^−1^ of MC-RR was completely eliminated within 36 h by *Sphingopyxis* sp. USTB-05, and in 10 h by its cell-free extract (CE) with 350 mg L^−1^ of protein [41]. An initial MC-LR of 28.8 mg L^−1^ could be biodegraded completely within 3 h by the CE of *Sphingopyxis* sp. USTB-05 [42]. An MC-YR concentration of 14.8 mg L^−1^ was also completely eliminated within 10 h by the CE [43]. Recently, we further found that NOD could also be biodegraded by both *Sphingopyxis* sp. USTB-05 and its CE [44]. Two biodegradation products were observed by high performance liquid chromatography (HPLC), and further analyzed on a liquid chromatogram mass spectrum (LC-MS). We inferred that at least two enzymes participated in the process of NOD biodegradation by *Sphingopyxis* sp. USTB-05 [44].

Because NOD biodegradation by the whole strain of *Sphingopyxis* sp. USTB-05 is consecutive, it is difficult to ascertain that all the intermediate products were traced successfully. So, investigating the enzymes encoded by the biodegradation genes and controlling the biodegradation process step by step are very important for pathway clarification. However, the minimal information about the heterologous expression of the enzymes limits further studies on the mechanism of NOD biodegradation. In this study, we show that the enzyme USTB-05-A converted cyclic NOD into its linear type as the first product by hydrolyzing the Arg–Adda peptide bond. USTB-05-C then cut off the Adda–Glu peptide bond of linearized NOD and produced Adda as the second product. Based on the successful cloning and expression of the *USTB-05-A* gene from *Sphingopyxis* sp. USTB-05 and homology modeling of enzyme USTB-05-A in previous works [45], site-directed mutants of *USTB-05-A* were constructed in this paper. Seven crucial sites for the first recombinant enzyme USTB-05-A were founded by comparing the enzyme activities of recombinant USTB-05-A and its mutants.

## 2. Results

### 2.1. Enzymatic Activity Detection of Recombinant CEs

**Figure 1:**
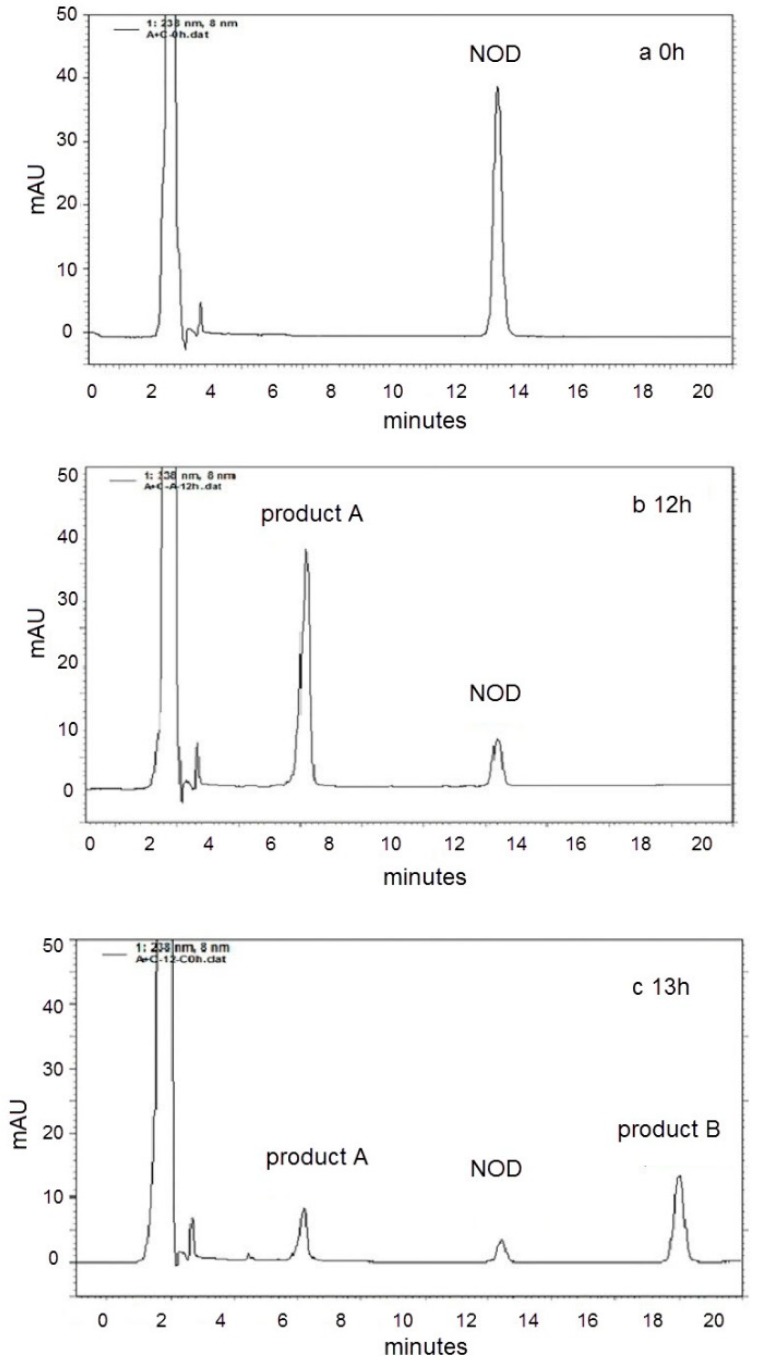

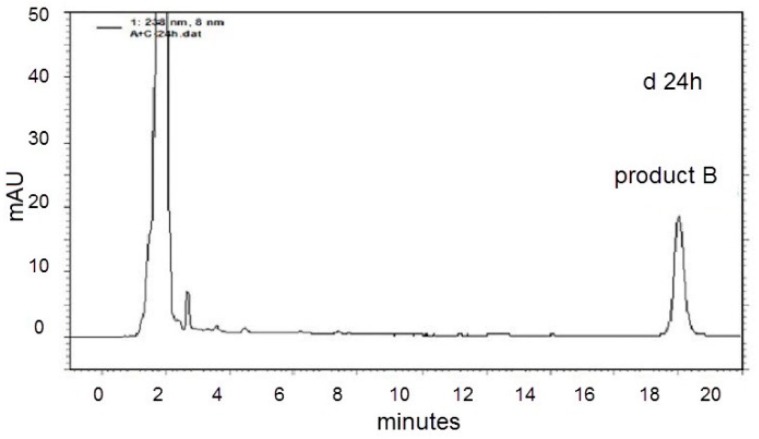
HPLC profiles for nodularin (NOD) biodegradation by cell-free extracts (CEs) of recombinant pGEX-4T-1/*USTB-05-A/*BL21(DE3) and pET30a(+)/*USTB-05-C/*BL21(DE3) after the following times: (**a**) 0 h; (**b**) 12 h; (**c**) 13 h; (**d**) 24 h.

**Figure 2:**
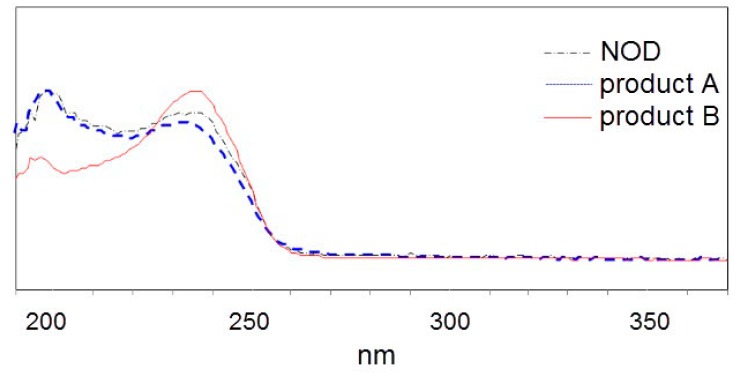
The UV spectra profiles of NOD and its two products.

For treatment AC, the CE containing crude USTB-05-A protein was added to phosphate buffered saline (PBS) containing NOD at 0 h, and then the CE containing crude USTB-05-C protein was added to the above solution at 12 h. In this treatment, the initial NOD (13.7 mg L^−1^) is biodegraded by CE from pGEX-4T-1/*USTB-05-A/*BL21(DE3) recombinant bacteria containing 158 mg L^−1^ protein. In Figure 1a,b, the retention time of NOD was 13.46 min. The new peak of product A at the retention time of 7.26 min was highly increased at 12 h with a decrease of NOD. At 12 h, the CE of recombinant pET30a(+)/*USTB-05-C/*BL21(DE3) containing 165 mg L^−1^ of protein was added. A new peak of product B at the retention time of 19.03 min appeared at 13 h and increased continuously until 24 h, while the peak of product A correspondingly decreased and disappeared (Figure 1c,d). The ultraviolet (UV) spectra of peak A and B were similar to that of NOD in the wavelength range of 200 nm to 370 nm (Figure 2), suggesting that Adda remained in product A and B. For the control group of treatment AC with CE of recombinant pET30a(+)/BL21(DE3) without protein USTB-05-C, the concentration of product A was shown to be constant (Figure S1).

In the control group for treatment A with CE of recombinant pGEX-4T-1/BL21(DE3) without protein USTB-05-A, the concentration of NOD was shown to be constant (Figure S2). In treatment A with CE containing crude protein USTB-05-A, the results were similar to treatment AC during the first 12 h, but the peaks of product A and NOD remained constant and no other peaks appeared until 24 h. In the control group for treatment C with CE of the recombinant pET30a(+)/BL21(DE3) without protein USTB-05-C and treatment C group with CE containing crude protein USTB-05-C, the concentrations of NOD were shown to be constant (Figure S3). These results indicated that recombinant enzyme USTB-05-A is involved in the first step of NOD biodegradation, and recombinant enzyme USTB-05-C is involved in the second step.

### 2.2. LC-MS Analysis of NOD and Its Biodegradation Products

**Figure 3:**
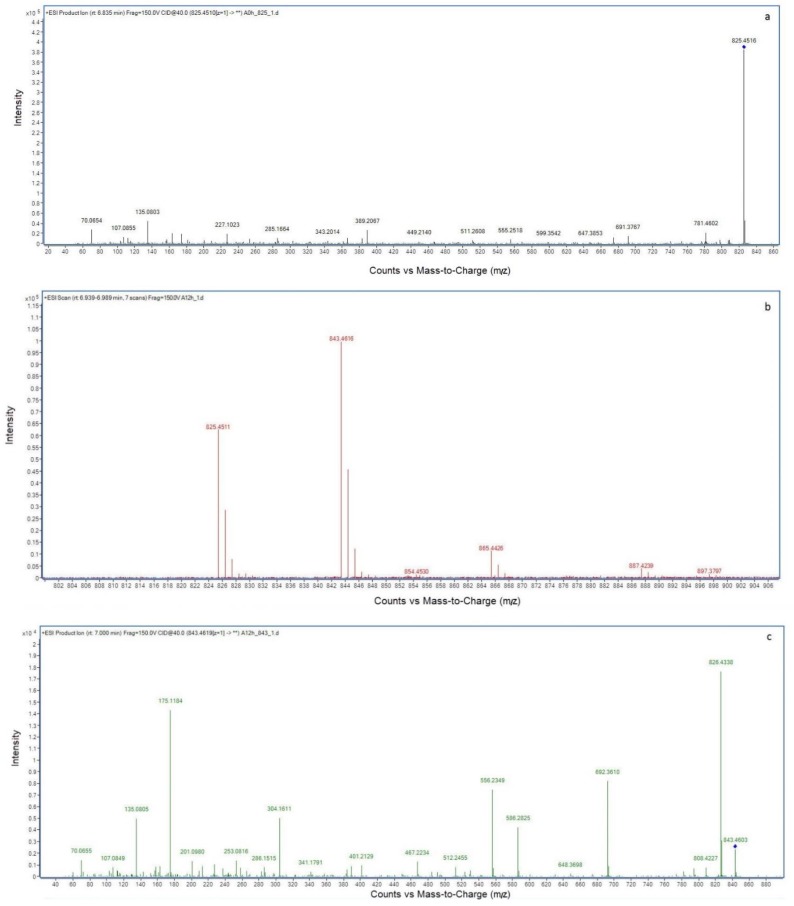

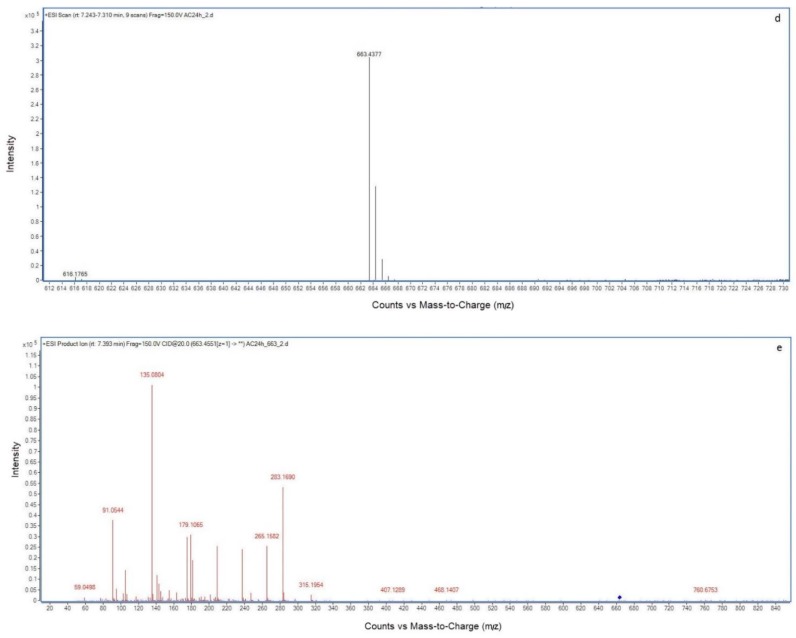
Liquid chromatogram mass spectrum (LC-MS) profile of NOD and its products. (**a**) MS spectrum for NOD; (**b**) MS spectrum for product A; (**c**) MS/MS spectrum for product A; (**d**) MS spectrum for product B; (**e**) MS/MS spectrum for product B.

The *m*/*z* ratios of NOD and its biodegradation products were measured by LC-MS/MS (Figure 3). The MS analysis of NOD in Figure 3a showed that the major ion (*m*/*z* 825.4516) was the protonated molecular ion of NOD [M+H]^+^. The molecular ion at *m*/*z* 843.4616 which was m/z 18 more than NOD indicated that product A was hydrolyzed NOD [M+H_2_O+H]^+^ (Figure 3b). In the fragmentation spectrum of the *m*/*z* 843.4616 ion [M+H_2_O+H]^+^ (Figure 3c), six main fragment ions were formed: 692.3610 [M+H_2_O−151+H]^+^ 586.2825 [CH_3_CH_2_CHO-Glu-Mdhb-MeAsp-Arg-OH+2H]^+^, 556.2349 [CO-Glu-Mdhb-MeAsp-Arg-OH]^+^, 304.1611 [MeAsp-Arg-OH+2H]^+^, 175.1184 [Arg-OH+2H]^+^ and 135.0805 PhCH_2_CHOCH_3_. The ion [M+H_2_O−151+H]^+^ (*m*/*z* 692.3610) was *m*/*z* 151 less than hydrolyzed NOD (*m*/*z* 843.4616), corresponding to the loss of the amino NH_2_ group (Molecular Weight (MW): 16) and the phenylethymethoxy group PhCH_2_CHOCH_3_ (*m*/*z* 135.0805) via random fragmentation. However, all bonds and ions could be found through major peak analyses, except for the Adda and Arg bond. This analysis showed that enzyme USTB-05-A hydrolyzed the Adda–Arg bond in the first step of NOD biodegradation, converting cyclic NOD to the linear type. The protonated product B was detected at *m*/*z* 663.4377 (Figure 3d), coinciding with the dimeric ion of Adda (MW: 331). In the MS/MS spectrum for product B (Figure 3e), the ions at *m*/*z* 315.1953 and *m*/*z* 135.0804 were generated through losing the amino NH_2_ group (MW: 16) and the PhCH_2_CHOCH_3_ group form Adda, respectively. The *m*/*z* 283.1690 [Adda−NH_3_−CH_3_OH+H]^+^, 91.0544 [PhCH_2_]^+^, 179.1065 [Adda−PhCH_2_CHOCH_3_−NH_3_+H]^+^ ions also confirmed the structure of Adda, which was inferred as product B.

### 2.3. Recombinant USTB-05-A and Its Mutants

**Figure 4:**
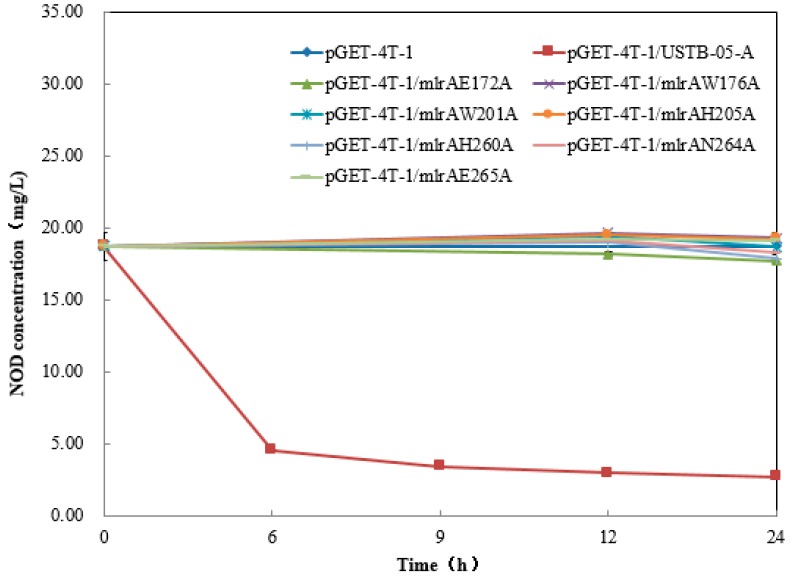
NOD biodegradation kinetics by CEs from *E. coli* BL21 (DE3) cells transformed with *USTB-05-A* and *USTB-05-A* mutants.

As shown by the NOD biodegradation kinetics in Figure 4, the USTB-05-A enzyme can degrade about 75% of 18.7 mg/L NOD within 6 h and 85% within 24 h. However, the activities of the mutants against NOD were all completely abolished (Figure 4), which confirmed that these seven sites were crucial for the USTB-05-A enzyme to biodegrade NOD.

## 3. Discussion

Biodegradation is essential for the reduction of NOD and MCs in water. Bourne et al. have detected the gene cluster for MC biodegradation, which includes *mlrA*, *mlrB*, *mlrC*, and *mlrD* from *Sphingomonas* sp. strain MJ-PV [27,35]. The enzyme MlrA encoded by the biodegradation gene *mlrA* is crucial for opening the cyclic structure of MCs by cleaving the Adda–Arg peptide bond. After, linearized MCs are biodegraded by the enzyme MlrB and Mlr C. Studies about biodegradation genes or pathways of MC degredation can be found, but less information is available on NOD, which is also a typical type of cyanobacterial toxin in natural water bodies [6,46]. Most of the literature emphasized the toxicity, toxin biosynthesis, detection methods of NOD and bacterial strains for NOD biodegradation. It has been shown that not only microbial populations [33,47,48], but also single strains [27,28,29] are able to biodegrade MCs, NOD or both. The bacterial strains in the *Sphingomonas* genus are the most reported strains to biodegrade MCs. The promising strain *Sphingopyxis* sp. USTB-05 has a strong ability to biodegrade MCs, and has at least three enzymes encoded by biodegradation genes [31,49]. *Sphingopyxis* sp. USTB-05 was also found to have the ability to biodegrade NOD and two biodegradation products were observed by HPLC at a wavelength of 238 nm. The pathway for NOD biodegradation by *Sphingopyxis* sp. USTB-05 was also speculated [44] but not entirely clear as biodegradation by the whole strain of *Sphingopyxis* sp. USTB-05 was a consecutive process, not step by step.

**Figure 5:**
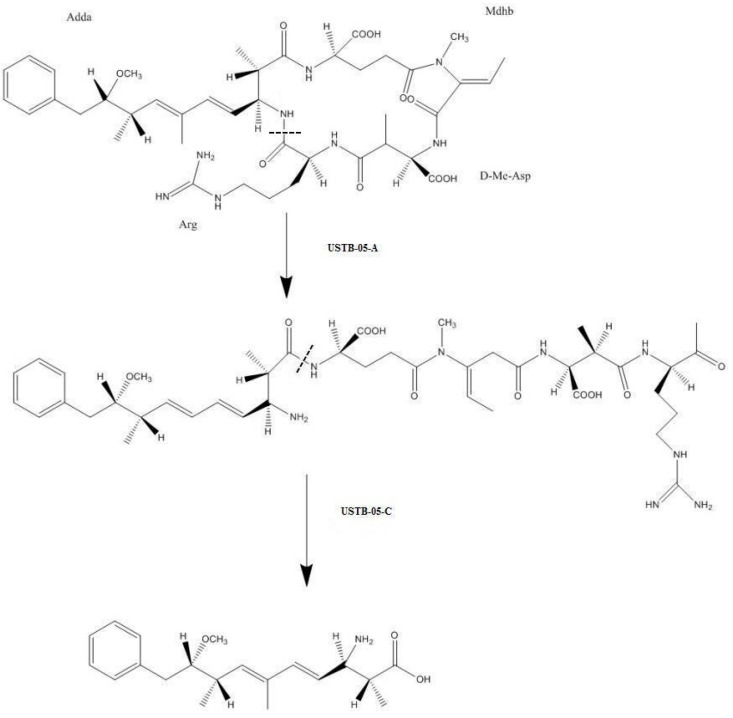
The suggested pathway for biodegrading NOD by recombinant enzymes USTB-05-A and USTB-05-C.

With the cloned *USTB-05-A* and *USTB-05-C* we previously studied on MCs [30], the functions of the USTB-05-A and USTB-05-C enzymes were evaluated for NOD biodegradation (Figure 1 and Figure 2). The results showed that the USTB-05-A enzyme was active in the first step of NOD biodegradation, and the USTB-05-C enzyme participated in further biodegradation from the second step. The USTB-05-A enzyme hydrolyzed cyclic NOD (*m*/*z* 825.4516) (Figure 3a) into product A (*m*/*z* 843.4616) (Figure 3b). Based on the analysis of the MS/MS spectrum for product A (Figure 3c), it could be inferred that the USTB-05-A enzyme broke the Adda–Arg bond in the ring of NOD through the process of adding one hydrogen on the NH_2_ group of Adda and linking one hydroxyl on the carboxyl group of arginine. The linearized NOD was the product in the first step of NOD biodegradation (Figure 5). The result was similar to that reported by Kato et al.: nodularin cleaved at the Arg–Adda bond by strain B-9 [50]. The Arg–Adda bond break also was the first step for MC biodegradation [27]. Product B (*m*/*z* 663.4377) in the second step was observed as the dimeric ion of Adda (MW: 331), which was due to the catalysis of the linearized NOD by the USTB-05-C enzyme (Figure 3d). A previous report [51] showed the same result for the dimeric ion of Adda (*m*/*z* 663). However, for the formation of dimeric Adda and the rest of the molecule, there were no explanations in the literature. With the MS/MS spectrum for the second product (Figure 3e), it could be inferred that the recombinant enzyme USTB-05-C cut off the Adda–Glu band of linearized NOD, producing Adda as the product in the second step of biodegradation (Figure 5).

Based on our previous work on the cloning of *USTB-05-A* (also known as *mlrA*) in *Sphingopyxis* sp. USTB-05 [30] and homology modeling of the USTB-05-A enzyme, seven mutants were constructed. Briefly speaking, the structure for the USTB-05-A enzyme was predicted with eight transmembrane α-helices (TM1–TM8). Following the template of MmRce1 with an active site of H^260^AIH^263^NE^265^, USTB-05-A was assumed to bear an abortive infectivity (ABI) domain composed of four transmembrane helices TM4–TM7. The ABI domain was surrounded by the other four helices, which together form a large cavity accessible to substrates such as NOD. The critical catalytic residues (E140, H173, H227 and N231) of MmRce1 were found to be well conserved in the USTB-05-A enzyme (E172, H205, H260, and N264). Two conserved Trp residues of W144 and W169 in MmRec1 (W176 and W201 in USTB-05-A) were found to face the carboxylate side chain of E140 (E172 in USTB-05-A) for a better activation of catalytic water. Then, these residues were verified by following docking- and site-directed mutation experiments [45]. By comparing the enzyme activities of recombinant USTB-05-A and its mutants, seven crucial sites (E172, W176, W201, H205, H260, N264, and E265) for the activity of the USTB-05-A enzyme were found. For MC-LR biodegradation kinetics in our previous study, the point mutants E172A, H205A, and H260A resulted in a complete loss of enzymatic activity. W176A and W201A mutants led to a decrease in the reaction rate to some extent and N264A results in a significant decrease in MlrA activity [45]. However, for the NOD biodegradation kinetics in this study, all seven mutants were completely inactivated. The results showed that these seven sites were crucial for MC-LR biodegradation, and even more important for NOD biodegradation. 

USTB-05-A is likely a glutamate protease belonging to type II CAAX prenyl endopeptidases. Combined with the biodegradation of NOD by USTB-05-A and its mutants, a complete enzymatic mechanism for NOD biodegradation by USTB-05-A is proposed: Glu172 and His205 activate a water molecule facilitating a nucleophilic attack on the Adda–Arg peptide bond of NOD; Trp176 and Trp201 contact the carboxylate side chain of Glu172 and accelerate the reaction rates; and His260 and Asn264 function as an oxyanion hole to stabilize the transition states. 

## 4. Conclusions

The enzymatic pathway for biodegrading nodularin by *Sphingopyxis* sp. USTB-05 was clarified in this study. The enzyme USTB-05-A converted cyclic NOD into its linear type as the first product by hydrolyzing the Arg–Adda peptide bond, and USTB-05-C cut off the Adda–Glu peptide bond of linearized NOD and produced Adda as the second product. A complete enzymatic mechanism for NOD biodegradation by USTB-05-A was also proposed. The active site Glu172 and His205 activated a water molecule, facilitating a nucleophilic attack on the Adda–Arg peptide bond of NOD. Trp176 and Trp201 contacted the carboxylate side chain of Glu172 and accelerated the reaction rate. His260 and Asn264 functioned as an oxyanion hole to stabilize the transition states. The findings provide good foundations for investigating the biodegradation mechanism of both MCs and NOD in the future.

## 5. Materials and Methods 

### 5.1. Bacterial Strains, Culture Conditions and Reagents

Recombinant pGEX-4T-1/*USTB-05-A/*BL21(DE3) and pET30a(+)/*USTB-05-C/*BL21(DE3) were previously studied on MCs and used in this study for NOD biodegradation [30,49]. *E. coli* TOP10 and *E. coli* BL21(DE3) were purchased from Sangon Biotech (Shanghai, China). The recombinant and host strains were cultured in Luria–Bertani (LB) medium [41] on a shaker at 200 rpm at an appropriate temperature. The vectors pGEX-4T-1 and pET30a(+), restriction enzymes *Bam*HI, *Xho*I, *Sac*I and *Not*I, the plasmid mini-prep kit and the polymerase chain reaction (PCR) kit were obtained from Sangon Biotech (Shanghai, China). Standard NOD was purchased from Enzo Science Inc., Farmingdale, NY, USA. All other chemicals were analytical grade. 

### 5.2. Expression of Gene USTB-05-A and USTB-05-C

The expression of recombinants was performed as previously described [30,49]. The confirmed clones of pGEX-4T-1/*USTB-05-A/*BL21(DE3) and pET30a(+)/*USTB-05-C/*BL21(DE3) were grown in LB medium with 100 μg mL^−1^ ampicillin or 50 μg mL^−1^ kanamycin for 3 h, respectively. The inoculated culture was grown at 37 °C and 200 rpm to an optical density at 600 nm (OD_600nm_) of 0.6, induced with isopropyl-β-D-thiogalactoside (IPTG) at a final concentration of 0.1 mmol L^−1^ and incubated at 30 °C for 3 h. The flask culture was grown in the same conditions above with an incubation amount of 10% and culture volume of 50 mL per 500 mL shake flask. One liter of recombinant cell culture was harvested by centrifugation (12,000 g, 4 °C, 25 min), washed thrice with PBS (50 mM, pH 7.3) and resuspended in 20 mL PBS to a final concentration of 20–30 g dry cell weight per liter. The cells were disrupted by sonication (400 W, 20 min) in an ice bath, and centrifuged (15,000 rpm, 4 °C, 30 min). The supernatant was collected as CE. Protein content was determined according to Bradford [52].

### 5.3. Enzymatic Activity of Recombinant USTB-05-A and USTB-05-C

The experiments were carried out with five treatments as following:

- Control groups for treatment A and treatment C: the CEs of recombinant bacteria pGEX-4T-1/BL21(DE3) without protein USTB-05-A and recombinant bacteria pET30a(+)/BL21(DE3) without protein USTB-05-C were added into two tubes with PBS containing NOD, respectively.
- Treatment A: the CE of recombinant bacteria pGEX-4T-1/*USTB-05-A*/BL21(DE3) containing crude protein USTB-05-A was added into PBS containing NOD.
- Treatment C: the CE of recombinant bacteria pET30a(+)/*USTB-05-C*/BL21(DE3) containing crude protein USTB-05-C was added into PBS containing NOD.
- Treatment AC: at 0 h, the CE containing crude protein USTB-05-A was added into PBS containing NOD. At 12 h, the CE containing crude protein USTB-05-C was added to the above solution.
- Control group for treatment A and treatment C: at 0 h, the CE containing crude protein USTB-05-A was added into the test tubes with PBS containing NOD. At 12 h, the CE of pET30a(+)/BL21(DE3) without protein USTB-05-C was added to the above solution.

Table 1 shows these experiment conditions in brief.

In each reaction system of 4 mL, the initial concentration of NOD was 13.7 mg L^−1^. The protein concentrations of CEs containing USTB-05-A and USTB-05-C were 158 mg L^−1^ and 165 mg L^−1^, respectively. All test tubes were placed in the incubator at 30 °C with shaking at 200 rpm. Samples of 200 μL were taken at 0 h, 3 h, 12 h, 13 h, 18 h and 24 h, respectively. Concentrated hydrochloric acid (2 µL) was added immediately to stop the reaction. Samples were centrifuged (15,000 rpm, 2 min) and the undiluted supernatants were taken for the detection by HPLC. 

For product identification, samples at several reaction time points were taken and purified through the C_18_ solid-phase extraction cartridge (Waters, OASIS™ HLB, USA). Methanol (40 μL) was added at a rate of 1 mL min^−1^. The treated sample was collected and analyzed by liquid chromatography tandem-mass spectrometry (Agilent 6530 Accurate-Mass Q-TOF LC/MS, Agilent Technologies Inc., Wilmington, DE, USA) to determine the *m*/*z* ratios of NOD and the biodegradation products.

### 5.4. Site-Directed Mutagenesis and Enzymatic Activity Comparison

Based on our previous work involving the cloning of *USTB-05-A* (also known as *mlrA*) in *Sphingopyxis* sp. USTB-05 [30] and homology modeling of enzyme USTB-05-A, site-directed mutagenesis for the construction of seven mutants including *mlrA*E172A, *mlrA*W176A, *mlrA*W201A, *mlrA*H205A, *mlrA*H260A, *mlrA*N264A, and *mlrA*E265A was performed by overlap extension PCR as previously reported [45]. The expression of recombinant *USTB-05-A* mutants and preparation of CEs were in accordance to the steps for recombinant USTB-05-A mentioned above. For enzymatic activity comparison of recombinant USTB-05-A and mutants, the initial concentration of NOD was 18.7 mg L^−1^ and the protein concentration of CEs was 350 mg L^−1^. Samples of 200 μL were taken at 0 h, 6 h, 9 h, 12 h and 24 h, respectively.

### 5.5. Analysis Methods

The concentration of NOD was detected by high performance liquid chromatography (HPLC) (LC10ATVP, Shimadzu Co., Ltd., Tokyo, Japan) with a UV diode array detector at 238 nm. An Agilent TC–C_18_ column (4.6 × 250 mm) (Agilent, 1200 series, Wilmington, DE, USA) was used. Chromatographic grade acetonitrile and water containing 0.05% (*v*/*v*) trifluoroacetic acid (35:65, *v*/*v*) was used as the mobile phase. The flow rate was 1.0 mL min ^-1^, and the injection amount was 20 μL. The spectra of NOD and its biodegradation products were detected at the time of HPLC analysis.

For MS detection, an analytical column OOD-4622-ECLC (100 mm × 4.6 mm i.d., 2.6 μm, Phenomenex Inc., Torrance, CA, USA) was used at a maintained temperature of 30 °C. Precursor ions for samples and internal standards were determined from mass spectrum obtained during infusion into the Agilent 6530 Accurate-Mass Q-TOF LC/MS. An electrospray ionization (ESI) source was used. The MS detection conditions were: capillary voltage of 4000 V, nebulizer gas (N_2_) pressure of 35 psi, desolvation gas flow rate of 10 L min^−1^ and desolvation gas temperature of 300 °C.

## Figures and Tables

**Table 1:** Experiment conditions of the treatments in brief.

Treatment	Experiment Condition
Control group for treatment A	NOD + the CEs of pGEX-4T-1/BL21(DE3)*
Control group for treatment C	NOD + the CEs of pET30a(+)/BL21(DE3)**
Treatment A	NOD + USTB-05-A
Treatment C	NOD + USTB-05-C
Treatment AC	NOD + USTB-05-A, 12 h later, + USTB-05-C
Control group for treatment AC	NOD + USTB-05-A, 12 h later, + the CEs of pET30a(+)/BL21(DE3)

* pGEX-4T-1/BL21(DE3) is the empty vector control for recombinant bacteria pGEX-4T-1/*USTB-05-A* /BL21(DE3). ** pET30a(+)/BL21(DE3) is the empty vector control for recombinant bacteria pET30a(+)/*USTB-05-C*/BL21(DE3).

## Supplementary Materials

The following are available online at https://www.mdpi.com/2072-6651/11/10/549/s1, Figure S1: High performance liquid chromatography (HPLC) profiles for control group for treatment AC: the enzymatic biodegradation of Product A by CE of the control recombinant pET30a(+)/BL21(DE3) after the following times: (**a**) 0 h; (**b**) 12 h, Figure S2: High performance liquid chromatography (HPLC) profiles for control group for treatment A: the enzymatic biodegradation of NOD by CE of the recombinant pGEX-4T-1/BL21(DE3) after the following times: (**a**) 0 h; (**b**) 12 h, Figure S3: High performance liquid chromatography (HPLC) profiles for control group for treatment C: the enzymatic biodegradation of NOD by CE of the recombinant pET30a(+)/BL21(DE3) after the following times: (**a**) 0 h; (**b**) 12 h.
