# Supplementary material for: Cloning and Expression of Genes for Biodegrading Nodularin by Sphingopyxis sp. USTB-05
Source: Toxins (Basel). 2019 Sep 20;11(10):549. doi: 10.3390/toxins11100549 (PMC6832836; doi:10.3390/toxins11100549)
Supplement: Supplementary file 1 [file toxins-11-00549-s001.pdf]

Supplementary Materials: Cloning and Expression of Genes for Biodegrading Nodularin by Sphingopyxis sp. USTB-05

Qianqian Xu, Hongfei Ma, Jinhui Fan, Hai Yan, Haiyang Zhang, Chunhua Yin, Xiaolu Liu, Yang Liu and Huasheng Wang

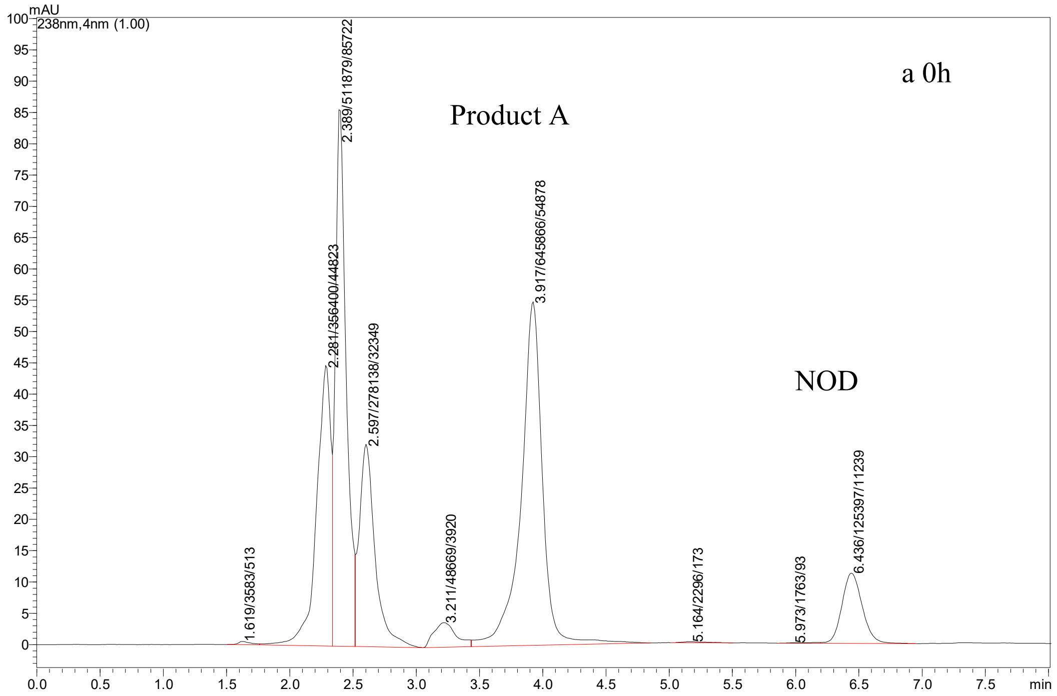

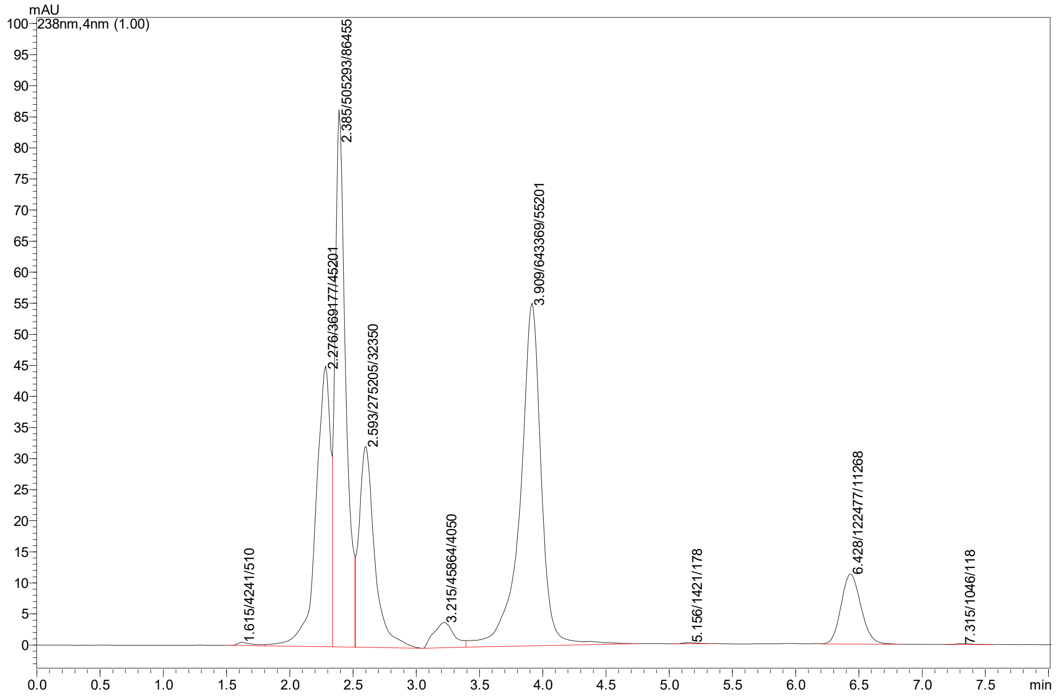

Figure S1. High performance liquid chromatography (HPLC) profiles for control group for treatment AC: the enzymatic biodegradation of Product A by CE of the control recombinant pET30a(+)/BL21(DE3) after the following times: (**a**) 0 h; (**b**) 12 h.

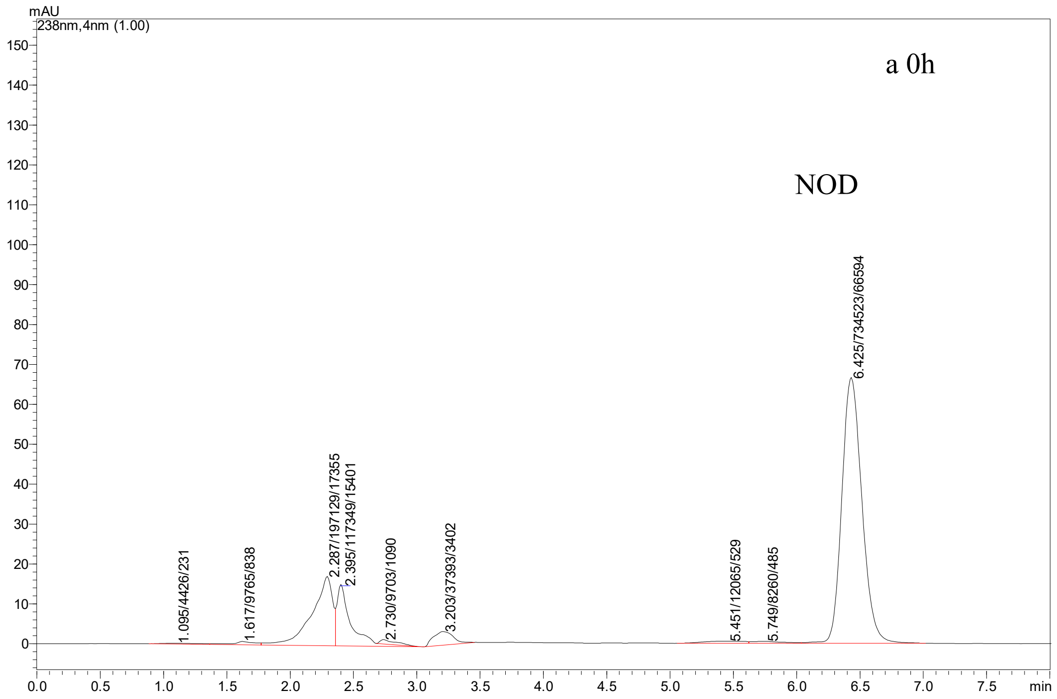

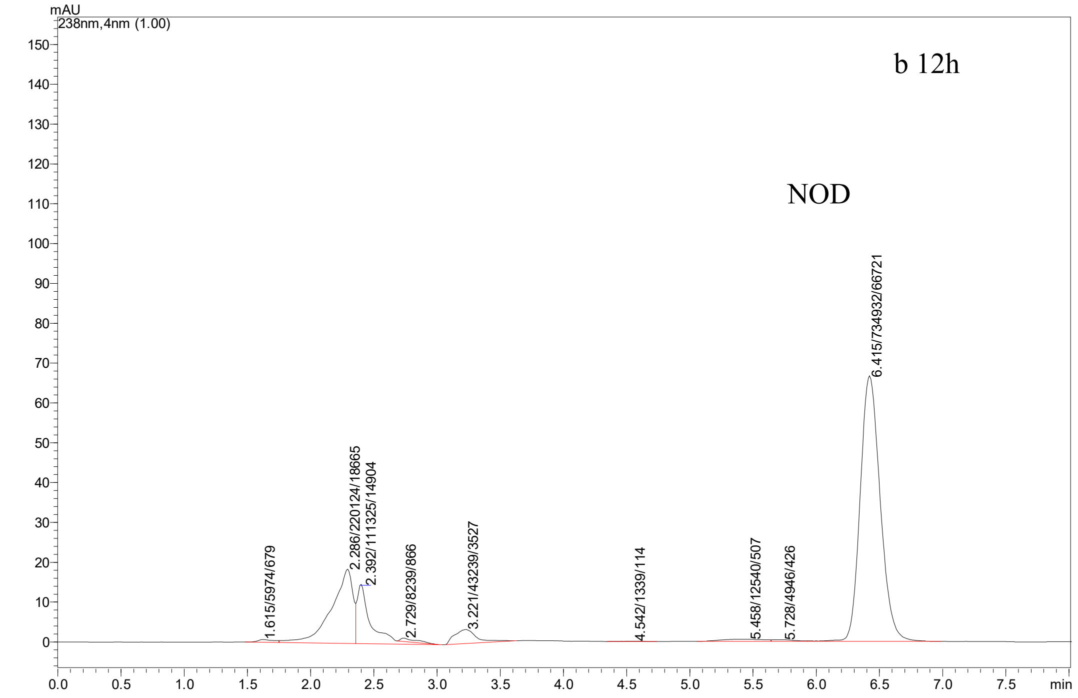

Figure S2. High performance liquid chromatography (HPLC) profiles for control group for treatment A: the enzymatic biodegradation of NOD by CE of the recombinant pGEX-4T-1/BL21(DE3) after the following times: (**a**) 0 h; (**b**) 12 h.

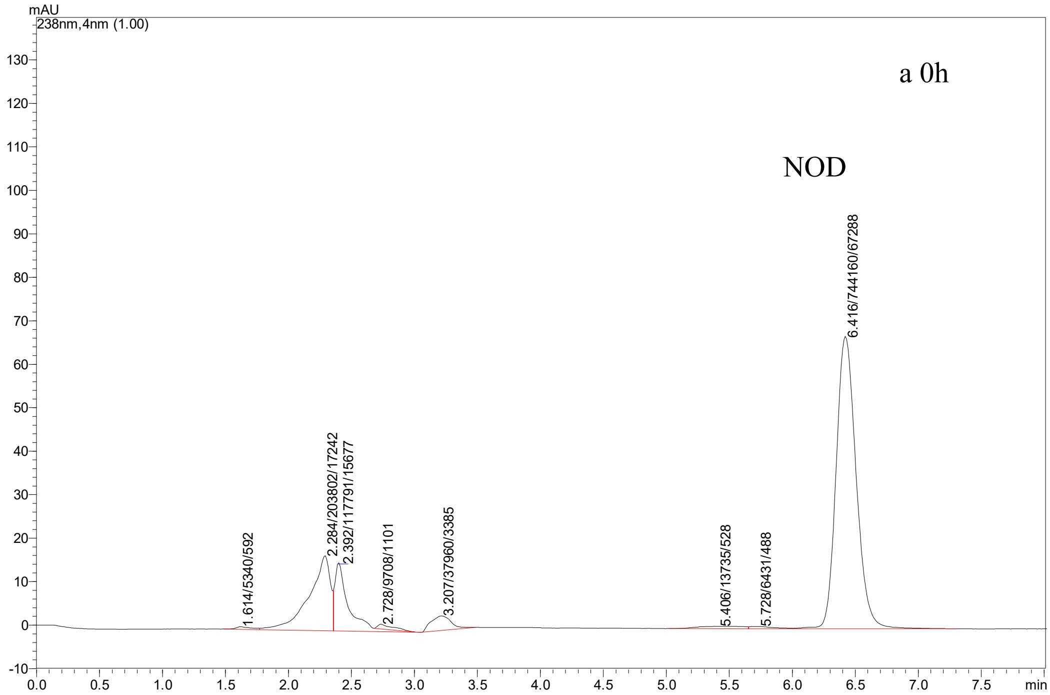

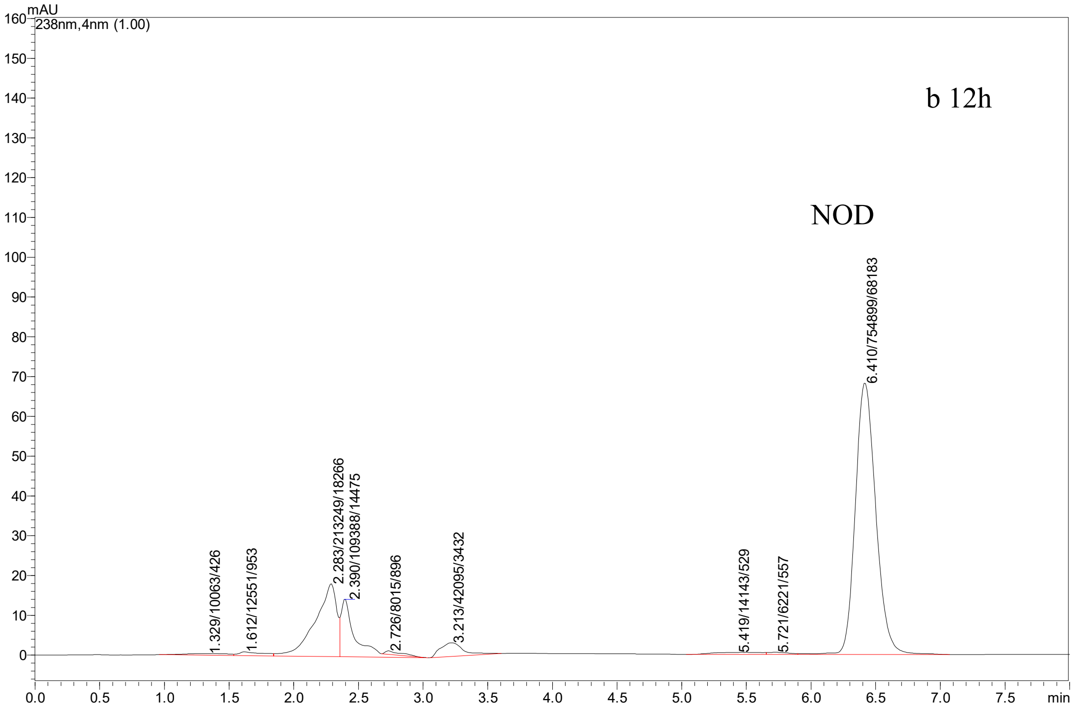

Figure S3. High performance liquid chromatography (HPLC) profiles for control group for treatment C：the enzymatic biodegradation of NOD by CE of the recombinant pET30a(+)/BL21(DE3) after the following times: (**a**) 0 h; (**b**) 12 h.
